# From Germline Variants to Tumor Outcome: GWAS‐Based Functional Genomics Prioritizes Colorectal Cancer Susceptibility Genes and Links *SMAD9* to Prognosis

**DOI:** 10.1155/humu/5617762

**Published:** 2026-08-20

**Authors:** Chengguang Hu, Guang Yang, Han Xiong, Yetong Xie, Zhao Yuan, Jiani Liu, Peiying Wang, Meng Li, Jiabao Xu, Qingyun Li, Chulan Li, Wenlin Han, Xin Peng, Li Zhang, Shanshan Cai, Fei Zhong, Lin Dai, Side Liu

**Affiliations:** ^1^ Department of Gastroenterology, Nanfang Hospital, Southern Medical University, Guangzhou, China, fimmu.com; ^2^ Department of Gastroenterology, Guangzhou Medical University Affiliated Women and Children′s Medical Center, Guangzhou, China; ^3^ Department of General Surgery, Guangzhou Twelfth People′s Hospital, Guangzhou Medical University, Guangzhou, China, gzhmc.edu.cn; ^4^ Department of Traditional Chinese Internal Medicine, School of Traditional Chinese Medicine, Southern Medical University, Guangzhou, Guangdong, China, fimmu.com; ^5^ Department of Rheumatology and Immunology, Nanfang Hospital, Southern Medical University, Guangzhou, Guangdong, China, fimmu.com; ^6^ The First School of Clinical Medicine, Southern Medical University, Guangzhou, Guangdong, China, fimmu.com; ^7^ The second Affiliated Hospital of Guilin Medical University, Guilin, Guangxi, China, glmc.edu.cn; ^8^ The Second School of Clinical Medicine, Southern Medical University, Guangzhou, Guangdong, China, fimmu.com; ^9^ Department of Nursing, Jiuquan Hospital of Shanghai General Hospital (Jiuquan People′s Hospital), Jiuquan, China; ^10^ Division of Biomedical and Life Sciences, Faculty of Health and Medicine, Lancaster University, Lancaster, Lancashire, UK, lancs.ac.uk; ^11^ Department of Infectious Diseases, Nanfang Hospital, Southern Medical University, Guangzhou, China, fimmu.com

**Keywords:** AlphaGenome, causal transcriptome-wide association study, colorectal cancer, expression quantitative trait locus (eQTL), genome-wide association study (GWAS), germline variants, single nucleotide polymorphism (SNP)

## Abstract

**Introduction:**

Genome‐wide association studies (GWAS) have identified over 200 germline risk loci for colorectal cancer (CRC), yet the causal variants and genes behind most GWAS signals remain unknown and the link between inherited risk and tumor outcome is largely unexplored. Connecting germline single nucleotide polymorphisms (SNPs) to gene expression through expression quantitative trait loci (eQTL) and to clinical outcome is needed to interpret this inherited risk.

**Methods:**

We combined CRC GWAS summary data (73,149 cases and 112,467 controls of European ancestry) with GTEx v8 eQTL from colon sigmoid, colon transverse, small intestine terminal ileum, and whole blood, integrating causal transcriptome‐wide association study (cTWAS) with SuSiE fine‐mapping, Bayesian colocalization, MAGMA SNP‐to‐gene analysis, and AlphaGenome variant‐effect prediction. Prioritized proteins were assessed by western blot. Four experimentally selected regulatory variants were genotyped in HCT116, SW480, RKO, and NCM460 cells; genotype‐protein associations were tested across cell‐line means, and cis‐regulatory effects were assessed by allele‐specific expression (ASE) and reference‐versus‐alternate dual‐luciferase assays. Prognostic relevance was evaluated in TCGA colorectal tumors.

**Results:**

cTWAS identified seven genes with posterior inclusion probability (PIP) > 0.50, and colocalization across 78 gene‐tissue pairs revealed 20 associations with PP.H4 > 0.80. Four genes showed convergent evidence: SMAD9 (PIP = 0.916, PP.H4 = 0.981), MAP3K2 (PIP = 0.762, PP.H4 = 0.827), FADS1 (PIP = 0.632, PP.H4 = 0.942), and ACTR1B (PIP = 0.566, PP.H4 = 0.994). All four proteins were reduced in CRC cells. Alt‐allele dosage was inversely associated with SMAD9 (Pearson r = −0.985, BH‐adjusted *p* = 0.029) and FADS1 protein abundance (*r* = −0.995, BH‐adjusted *p* = 0.020), but not with MAP3K2 or ACTR1B. Reporter and ASE assays detected the clearest allele‐specific effects at SMAD9 and FADS1, whereas MAP3K2 and ACTR1B were null in the tested systems. Lower SMAD9 expression was nominally associated with poorer survival (log − rank *p* = 0.022 to 0.050), but these associations did not survive Benjamini–Hochberg correction across 12 tests.

**Conclusions:**

Integrating statistical genetics, regulatory prediction, and locus‐directed experiments prioritized SMAD9, MAP3K2, FADS1, and ACTR1B as CRC susceptibility genes. Functional evidence was strongest and most directionally coherent for SMAD9, demonstrated allele‐specific but context‐dependent regulation at FADS1, and placed experimental bounds on the proposed MAP3K2 and ACTR1B mechanisms.

## 1. Introduction

Colorectal cancer (CRC) is the third most commonly diagnosed malignancy and the second leading cause of cancer‐related mortality worldwide, with an estimated 1.93 million new cases and 930,000 deaths reported in 2022 [1, 2]. The global burden of CRC is projected to escalate substantially, with incidence forecasted to reach 3.2 million cases annually by 2040, driven by population aging, dietary transitions, and rising obesity prevalence in developing countries [3]. Twin and family studies have established that genetic factors account for approximately 35% of CRC risk based on the largest Nordic twin cohort to date [4], underscoring the imperative to identify heritable susceptibility determinants [4].

Over the past decade, GWAS have achieved remarkable progress in delineating the genetic architecture of CRC. The most comprehensive meta‐analysis to date, encompassing 100,204 cases and 154,587 controls of European and East Asian descent, has identified 205 independent germline risk associations, the majority of which are common single nucleotide polymorphisms (SNPs) located in noncoding regions of the genome [5]. Subsequent investigations have expanded this catalog to encompass associations across diverse ancestries and have begun to systematically prioritize functional variants and effector genes at established loci [6–9]. Despite these advances, the vast majority of GWAS signals reside in noncoding genomic regions, and the causal genes mediating these associations remain largely uncharacterized [10].

Transcriptome‐wide association studies (TWAS) have emerged as a powerful approach for connecting GWAS risk loci to biological mechanisms by integrating gene expression data with genetic association signals [11, 12]. By leveraging expression quantitative trait loci (eQTL) and germline SNPs as instrumental variables, TWAS methods such as PrediXcan and FUSION prioritize genes whose genetically regulated expression correlates with disease risk [11, 13]. However, conventional TWAS approaches are susceptible to confounding by linkage disequilibrium (LD), whereby noncausal genes in the vicinity of true causal genes may generate spurious associations [14]. The recently developed causal transcriptome‐wide association studies (cTWAS) method addresses this limitation by jointly modeling all genes and SNPs in a region using a Bayesian variable selection model with SuSiE fine‐mapping, thereby distinguishing genuine gene‐level effects from LD‐driven confounding [15].

Complementing TWAS approaches, Bayesian colocalization analysis provides orthogonal evidence for shared causal variants between GWAS and eQTL signals [16, 17]. The integration of colocalization with TWAS can substantially reduce false‐positive rates by confirming that the same genetic variant drives both disease risk and gene expression regulation within a given tissue context [18]. Furthermore, recent advances in deep learning genomics have enabled unprecedented resolution in predicting the functional consequences of noncoding genetic variants. AlphaGenome, developed by Google DeepMind, represents the current state‐of‐the‐art in variant effect prediction, jointly modeling chromatin accessibility, transcription, and three‐dimensional genome organization across hundreds of cell types to quantify the regulatory impact of individual nucleotide substitutions [19].

In this study, we integrated cTWAS, Bayesian colocalization, MAGMA gene‐level association testing, AlphaGenome variant‐effect prediction, and locus‐directed experiments to identify and functionally characterize CRC susceptibility genes from GWAS summary data and tissue‐specific eQTL. MAGMA was used as an orthogonal, model‐free cross‐check. The convergent loci were assessed at the protein level and, in response to the editor′s mechanistic recommendations, by cell‐line genotyping, genotype‐protein association, ASE, and reference‐versus‐alternate reporter assays. We further examined the prognostic relevance of the convergent genes in colorectal tumors while distinguishing inherited regulatory effects from tumor‐derived expression states.

## 2. Materials and Methods

### 2.1. GWAS Summary Statistics

CRC GWAS summary statistics were obtained from the European‐ancestry analysis comprising 73,149 cases and 112,467 controls (185,616 individuals) [5]. The dataset included 9,593,361 autosomal SNPs with effect allele, alternate allele, beta, standard error, Z‐score, and per‐SNP sample size. Coordinates were aligned to GRCh38/hg38.

### 2.2. cTWAS

The cTWAS analysis was performed using the cTWAS package as described by Zhao et al. [15]. This method jointly models SNPs and predicted gene expression to distinguish causal gene‐level effects from LD‐induced confounding. Expression prediction models were derived from GTEx v8 eQTL data [20] across four gastrointestinal‐relevant tissues, namely colon sigmoid, colon transverse, small intestine terminal ileum, and whole blood. These tissues were selected based on their direct relevance to CRC pathophysiology and the availability of adequate sample sizes for robust eQTL estimation. The cTWAS approach employs SuSiE for Bayesian fine‐mapping, yielding posterior inclusion probabilities (PIPs) for each gene and SNP in each region [21, 22]. Genome‐wide LD‐blocks were defined using the European‐ancestry block partitions distributed with the cTWAS package (approximately 1704 independent regions), and SuSiE fine‐mapping was run with the maximum number of single effects set to *L* = 5 per region. Genes with PIP > 0.50 were designated as significant cTWAS hits, consistent with established thresholds in the literature [15].

### 2.3. Bayesian Colocalization Analysis

Colocalization analysis was conducted using the coloc R package (Version 5.2) to assess whether CRC GWAS signals and tissue‐specific eQTL signals share a common causal variant [16, 17]. For each gene identified as significant in the cTWAS analysis and each of the four tissues examined, SNP‐level summary statistics were extracted within a 1‐Mb window centered on the gene body and subjected to the coloc.abf function with default priors (*p*1 = 1 × 10^−4^, *p*2 = 1 × 10^−4^, *p*12 = 1 × 10^−5^). These are the prior values recommended for genome‐wide eQTL–GWAS colocalization, whose sensitivity behavior has been characterized in detail by Wallace [18]. A total of 78 gene‐tissue pairs were tested. Strong colocalization evidence was defined as a posterior probability of hypothesis H4 (PP.H4) exceeding 0.80, indicating that both traits share a single causal variant.

### 2.4. MAGMA Gene‐Level Analysis

Gene‐level association testing was performed using MAGMA (Version 1.10) [23], which aggregates SNP‐level GWAS associations to gene‐level test statistics via a multimarker regression approach. SNPs were mapped to 19,892 protein‐coding genes using GENCODE v46 gene boundaries with a 10‐kb upstream and 0‐kb downstream window (MAGMA‐‐annotate window = 10.0); this upstream‐only window was applied deliberately to prioritize promoter‐proximal regulatory variation, a conservative choice that may under‐assign distal 3‐prime regulatory variants [24]. LD was estimated from the European‐ancestry subset of the 1000 Genomes Project Phase 3 reference panel [25]. Bonferroni correction was applied to determine genome‐wide significance (*p* < 2.51 × 10 − 6^−6^).

### 2.5. AlphaGenome Variant Effect Prediction

For each high‐confidence gene with convergent cTWAS and colocalization evidence, variant effect prediction was performed using AlphaGenome [19], a deep learning model developed by Google DeepMind that predicts the effects of genetic variants on gene regulation at single‐nucleotide resolution. AlphaGenome accepts 1‐Mb genomic intervals centered on the variant of interest and jointly predicts reference and alternate allele signals for four epigenomic modalities, namely RNA‐seq transcription, cap analysis of gene expression (CAGE) transcription start site activity, ATAC‐seq chromatin accessibility, and DNase‐seq hypersensitivity, across tissue‐specific ontology terms matching sigmoid colon (UBERON: 0001159), transverse colon (UBERON: 0001157), and large intestine (UBERON: 0000059). CAGE predictions, for which colon‐specific tracks are not represented in the public AlphaGenome training output, were generated across all 546 available cell‐type tracks. We verified that variant scores at gastrointestinally relevant tracks (intestinal epithelial‐cell proxies and tracks concordant with sigmoid‐/transverse‐colon H3K27ac signal) followed the same direction and similar magnitude as the pan‐tissue summary, indicating that the pooled estimate is unlikely to be inflated by tissue‐mismatched signals (Table S1). Three‐dimensional chromatin contact maps (Hi‐C) were predicted across 28 cell types at 512 × 512 bin resolution (approximately 2 kb per bin) covering the 1‐Mb window. Variant effect significance was assessed using the AlphaGenome variant scorers recommended by the developers for each modality, the CenterMaskScorer for DNase‐seq, ATAC‐seq and CAGE (computing reference‐versus‐alternate signal differences within a 2001‐bp window centered on the variant) and the GeneMaskLFCScorer for RNA‐seq (log‐fold change of gene‐body expression aggregated over annotated exons), with quantile scores exceeding |0.90|designated as significant. The |0.90|cutoff corresponds to the upper‐ and lower‐decile tails of the empirical background distribution of common‐variant scores released with the AlphaGenome model and is the threshold recommended by the model developers for prioritizing high‐impact regulatory variants. To assess threshold sensitivity, we additionally retabulated the number of significantly perturbed tracks at |0.80|and |0.95|quantile cutoffs. The relative ranking of the four lead variants and their gene‐specific modality patterns were preserved across all three thresholds (Table S1), confirming that the reported perturbation percentages are not artifacts of a single cutoff [19]. Reference epigenomic annotations were obtained from ENCODE [26] and GENCODE v46 [24] for gene structure annotations.

### 2.6. Western Blot Analysis

To assess the prioritized proteins, western blot analysis was performed for SMAD9, MAP3K2, FADS1, and ACTR1B in the CRC cell lines HCT116, SW480, and RKO and in NCM460 colonic epithelial cells. Total protein was extracted in RIPA buffer supplemented with protease and phosphatase inhibitors. Equal amounts of protein (30 *μ*g per lane) were separated by 10% SDS‐PAGE and transferred to PVDF membranes. Membranes were incubated with antibodies against SMAD9 (ab115900, Abcam), MAP3K2 (#19607, CST), FADS1 (10627‐1‐AP, Proteintech), ACTR1B (14076‐1‐AP, Proteintech), and GAPDH (#5174, CST), followed by the corresponding horseradish‐peroxidase‐conjugated secondary antibodies (#7074 and #7076, CST; 1:5000). Chemiluminescence images were acquired within the nonsaturating exposure range and quantified with ImageJ. Three independent Western blot experiments were performed for each cell line. Cell lines were treated as distinct biological models rather than as replicate observations. Target‐protein abundance was normalized to GAPDH, and each CRC line was compared separately with NCM460. Normality and variance assumptions were assessed before two‐sided Student′s or Welch′s *t* tests, as appropriate, with Benjamini–Hochberg correction across the four target proteins.

### 2.7. Cell‐Line Genotyping and Genotype‐Protein Association

**Figure 1:**
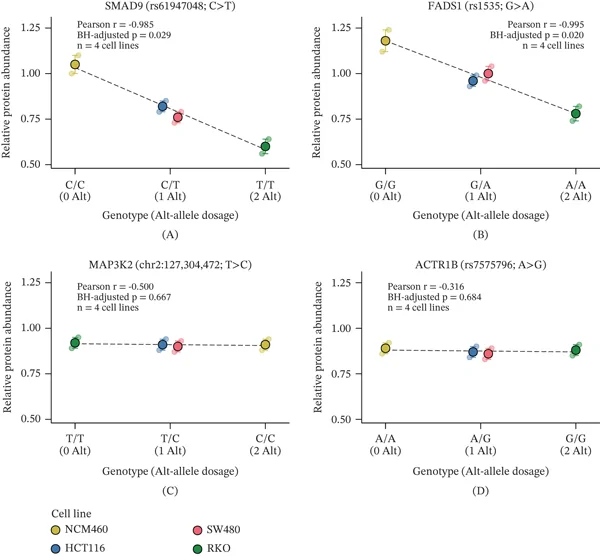
Cell‐line genotype‐protein associations at four experimentally assayed regulatory variants. (A) SMAD9 rs61947048 C>T, (B) FADS1 rs1535 G>A, (C) MAP3K2 chr2:127,304,472 T>C, and (D) ACTR1B rs7575796 A>G. Small translucent points show three Western blot measurements for each cell line; large outlined points and error bars show mean plus or minus SD. Dashed lines show the least‐squares trend across the four cell‐line means. Pearson correlations use alternate‐allele dosage (0, 1, or 2) and *n* = 4 cell‐line means; individual blot measurements were not treated as independent genotype observations. *P* values are Benjamini–Hochberg adjusted across the four genes.

Genomic DNA from HCT116, SW480, RKO, and NCM460 cells was amplified across four experimentally selected regulatory variants: SMAD9 rs61947048 (C>T), FADS1 rs1535 (G>A), MAP3K2 chr2:127,304,472 (T>C), and ACTR1B rs7575796 (A>G). Variant‐spanning amplicons were bidirectionally Sanger sequenced and genotypes were assigned as Ref/Ref, Ref/Alt, or Alt/Alt. The assayed variant is distinguished from the top association or AlphaGenome‐scored variant where these differed within a locus. Genotypes were coded as 0, 1, or 2 alternate alleles. For each gene, Pearson correlation was calculated between alternate‐allele dosage and the mean normalized protein abundance of each cell line (*n* = 4 cell lines); *p* values were Benjamini–Hochberg adjusted across the four genes. The three western blot measurements are displayed in Figure 1 but were not treated as independent genotype observations. Genotypes and replicate‐level densitometry are reported in Table S8.

### 2.8. Dual‐Luciferase Reporter and Allele‐Specific Expression Assays

**Figure 2:**
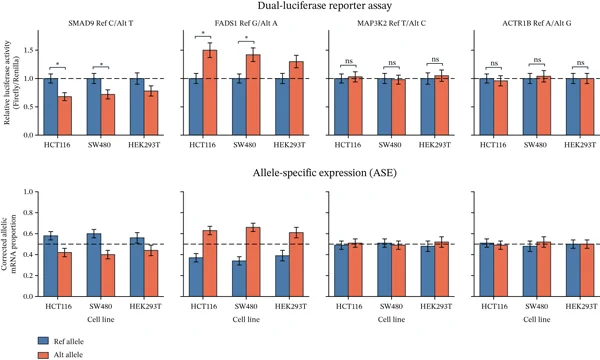
Allele‐specific reporter activity and expression at four CRC susceptibility loci. (A) Dual‐luciferase activity of reference and alternate constructs in HCT116, SW480, and HEK293T cells. Firefly signal was normalized to *Renilla* and to the reference construct within each cell line. (B) Corrected reference and alternate allelic mRNA proportions from ASE assays; the dashed horizontal line marks equal expression (0.50). Data are mean plus or minus SD from three independent experiments. ∗*p* < 0.05; ns, not significant.

For the reporter assay, approximately 500–1000‐bp regulatory fragments centered on each assayed variant were generated in reference and alternate forms and sequence verified. Enhancer‐like SMAD9 and ACTR1B fragments were tested in pGL3‐Promoter, which supplies an SV40 minimal promoter, whereas promoter‐like FADS1 and MAP3K2 fragments were tested in promoterless pGL3‐Basic. Reference and alternate constructs were transfected into HCT116, SW480, and HEK293T cells together with a *Renilla* control. Firefly signal was normalized to *Renilla* and then to the reference construct within each cell line. Three independent transfections were summarized as mean plus or minus SD; nominal two‐sided within‐cell‐line comparisons are denoted by ∗*p* < 0.05 or ns in Figure 2A.

ASE was evaluated in heterozygous assay‐eligible cells using variant‐spanning genomic‐DNA and cDNA amplicons. Allelic peak ratios in cDNA were normalized to the corresponding genomic‐DNA ratio to reduce amplification and base‐calling bias, and results are reported as corrected reference and alternate mRNA proportions, for which 0.50 denotes equal allelic expression. HCT116 and SW480 were the prespecified CRC models; HEK293T was included as an additional comparator. Three independent RNA preparations were summarized as mean plus or minus SD. The reporter and ASE summary values are provided in Table S9, and assay design and cross‐assay concordance are shown in Figure S2.

### 2.9. Multimethod Evidence Integration

To identify high‐confidence CRC susceptibility genes, evidence tiers were defined separately from the cross‐method convergence rule. Tier 1 required cTWAS PIP > 0.80 and colocalization PP.H4 > 0.80, whereas Tier 2 required PIP > 0.50 and PP.H4 > 0.50. For cross‐method prioritization, we used the joint criterion PIP > 0.50 and PP.H4 > 0.80; agreement between two independent frameworks was treated as a guard against LD‐driven false positives, and a PIP > 0.80 convergence threshold would have retained only SMAD9. Threshold sensitivity is reported in Table S6. Multiplicity was handled within each analytical layer: MAGMA *p* values were Bonferroni corrected across 19,892 genes, whereas cTWAS PIP and colocalization PP.H4 are Bayesian posterior quantities and were not assigned frequentist corrections. The convergence requirement was not described as a pooled multiple‐testing correction. Locus‐level visualization used custom LocusZoom‐style plots with LD estimated from the 1000 Genomes European reference panel using PLINK [27] and the standard r‐squared color scale.

### 2.10. Prognostic Association Analysis in TCGA

Prognostic relevance of the four convergent genes was assessed in the TCGA colorectal cohort (colon and rectal adenocarcinoma). Normalized RNA‐seq expression and curated overall survival, disease‐specific survival, and progression‐free interval endpoints for primary tumors were obtained from the UCSC Xena repository [28] and the TCGA Pan‐Cancer Clinical Data Resource [29]. For each gene, patients were divided at the median expression value, survival distributions were compared by the log‐rank test, and hazard ratios were estimated by Cox proportional‐hazards regression. Reported *p* values are nominal.

## 3. Results

### 3.1. cTWAS Identifies Seven Genes With Evidence of Causal Association With CRC Risk

**Figure 3:**
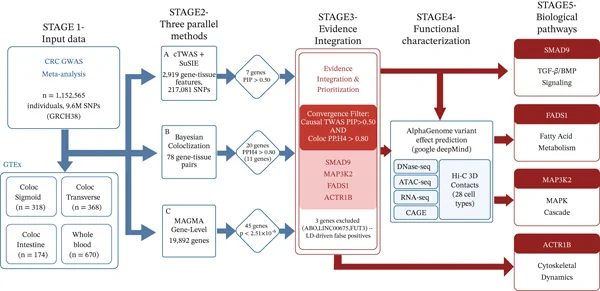
Study design and analytical workflow. Five‐stage integrative analysis for CRC susceptibility gene identification. Stage 1, input data from CRC GWAS meta‐analysis (73,149 cases and 112,467 controls of European ancestry, 9.6 M SNPs, GRCh38) integrated with GTEx v8 eQTL data from four gastrointestinal‐relevant tissues (colon sigmoid *n* = 318, colon transverse *n* = 368, small intestine terminal ileum *n* = 174, and whole blood *n* = 670). Stage 2, three parallel analytical methods, (A) cTWAS with SuSiE fine‐mapping identifying seven genes with PIP > 0.50, (B) Bayesian colocalization identifying 20 gene‐tissue pairs with PP.H4 > 0.80, and (C) MAGMA gene‐level analysis identifying 45 genome‐wide significant genes. Stage 3, evidence integration applying convergence filter (cTWAS PIP > 0.50 AND coloc PP.H4 > 0.80) to identify four dual‐significant genes (SMAD9, MAP3K2, FADS1, ACTR1B). Stage 4, AlphaGenome variant effect prediction across DNase‐seq, ATAC‐seq, RNA‐seq, CAGE, and Hi‐C modalities. Stage 5, biological pathway annotation.

**Figure 4:**
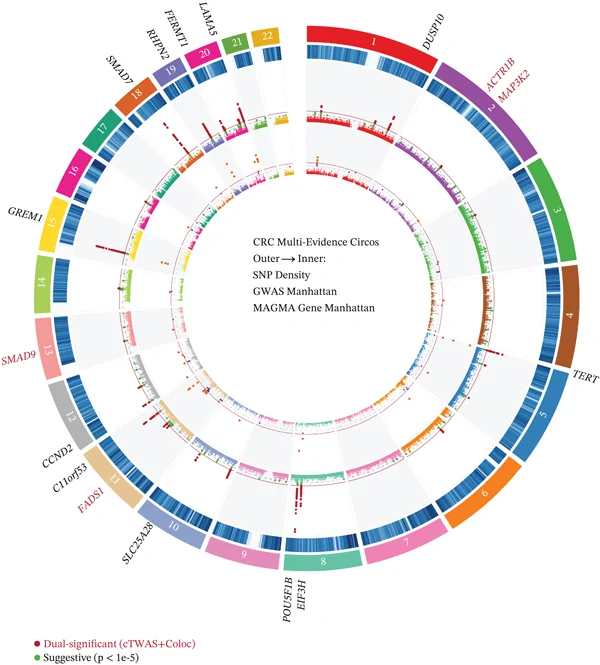
CRC multievidence circos plot. Genome‐wide visualization of CRC risk gene evidence. Tracks from outer to inner, SNP density, GWAS Manhattan plot, MAGMA gene‐level Manhattan plot. Dual‐significant genes (cTWAS PIP > 0.50 and Coloc PP.H4 > 0.80) and suggestive associations (*p* < 1 × 10^−5^) are highlighted. Key CRC susceptibility genes including SMAD9, MAP3K2, FADS1, and ACTR1B are labeled.

Application of cTWAS to CRC GWAS summary statistics across four gastrointestinal‐relevant tissues identified seven genes with PIP exceeding 0.50 (Figure 3). The strongest association was observed for *ABO* (PIP = 0.978), encoding the ABO blood group glycosyltransferase, followed by *LINC00675* (PIP = 0.957), a long noncoding RNA, and *SMAD9* (PIP = 0.916), a component of the BMP/TGF‐*β* signaling cascade. Additional significant genes included *MAP3K2* (PIP = 0.762), *FADS1* (PIP = 0.632), *ACTR1B* (PIP = 0.566), and *FUT3* (PIP = 0.562). Per‐tissue cTWAS Z‐scores for these seven genes are tabulated in Table S2. All four convergent genes (SMAD9, MAP3K2, FADS1, and ACTR1B) carried negative Z‐scores in every tissue in which they reached PIP > 0.10, indicating that genetically lowered expression is consistently associated with increased CRC risk, with no cross‐tissue direction reversal observed. Among these, *SMAD9* and *ABO* were detected across all four tissues (with PIP ranging from 0.71 to 0.92), whereas *LINC00675*, *FADS1*, and *FUT3* exhibited tissue‐restricted expression associations, suggesting context‐dependent regulatory mechanisms. A total of 2919 gene‐tissue expression features and 217,081 SNPs were jointly modeled, with 605 unique genes assigned to credible sets by the SuSiE fine‐mapping procedure (Figure 4).

### 3.2. Colocalization Analysis Confirms Shared Causal Variants for CRC Risk and Gene Expression

Bayesian colocalization analysis across 78 gene‐tissue pairs revealed 20 associations (25.6%) with strong colocalization evidence (PP.H4 > 0.80), involving 11 unique genes (Figure 5A). The strongest colocalization was observed for *ACTR1B* in the small intestine terminal ileum (PP.H4 = 0.994), with this gene demonstrating highly consistent colocalization across all four tissues (PP.H4 ranging from 0.963 to 0.994). *SMAD9* exhibited strong colocalization specifically in colon transverse tissue (PP.H4 = 0.981), whereas *FADS1* demonstrated significant colocalization in both colon sigmoid (PP.H4 = 0.942) and whole blood (PP.H4 = 0.896). Colon transverse tissue yielded the largest number of significant colocalizations (eight gene‐tissue pairs with PP.H4 > 0.80), followed by colon sigmoid (six pairs) and whole blood (five pairs).

Figure 5Regional association and CRC risk gene prioritization. (A) CRC risk gene prioritization heat map. Comprehensive multievidence summary for all prioritized genes, showing chromosome location, MAGMA −log₁₀(*p*), MAGMA FDR, cTWAS PIP, tissue‐specific detection (PIP > 0.1), cTWAS Z‐score, colocalization PP.H4, top tissue, and evidence classification (dual‐significant, supportive, or MAGMA‐only). Genes are grouped by evidence tier. (B) LocusZoom‐style regional association plots for the four dual‐significant loci (ACTR1B, FADS1, MAP3K2, and SMAD9). Each panel shows the SNP‐level − log₁₀(*p*) with LD *r* ^2^ color coding from 1000 Genomes European reference panel (top), tissue‐specific cTWAS gene PIP bar plots across four tissues (middle), and colocalization PP.H4 bars with the GENCODE gene track (bottom).

**Figure:**
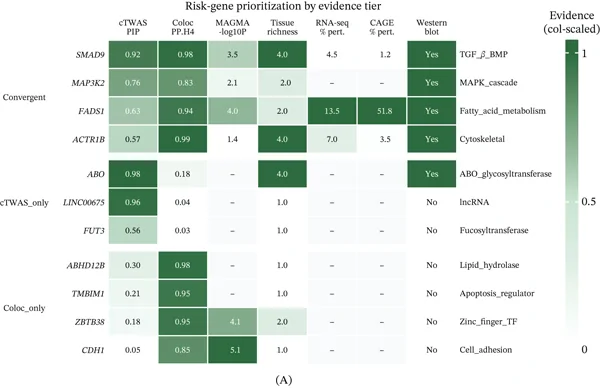
(A)

**Figure:**
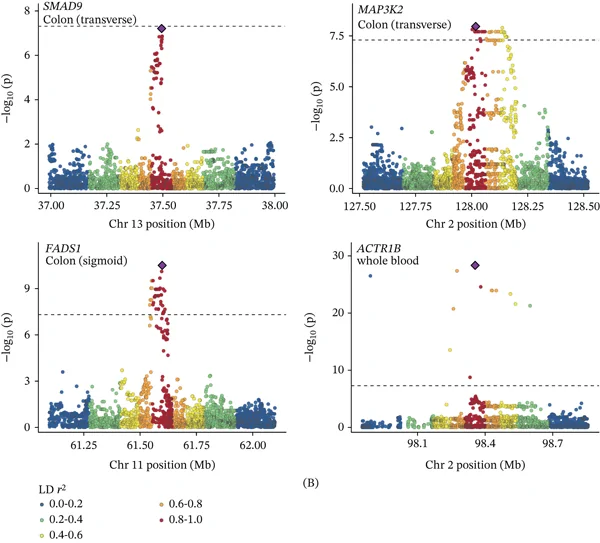
(B)

### 3.3. Convergent Evidence Identifies Four High‐Confidence CRC Susceptibility Genes

**Figure 6:**
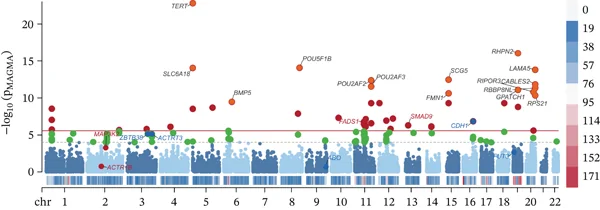
MAGMA gene‐level Manhattan plot with multimethod gene annotation. The *y*‐axis shows −log₁₀(*p*) from MAGMA gene‐level analysis across 19,892 protein‐coding genes. The red horizontal line indicates Bonferroni‐corrected genome‐wide significance (*p* < 2.51 × 10^−6^). A dashed gray line indicates suggestive significance. Genes are color‐coded by evidence category, with red for dual‐significant (cTWAS and colocalization), green for supportive cTWAS evidence, and blue for MAGMA‐only significance. The four convergent genes (SMAD9, MAP3K2, FADS1, and ACTR1B) and additional cTWAS‐significant genes (ABO, LINC00675, and FUT3) are labeled in red text. Top MAGMA‐significant genes are labeled in black.

Integration of cTWAS and colocalization identified SMAD9 (PIP = 0.916, PP.H4 = 0.981), MAP3K2 (PIP = 0.762, PP.H4 = 0.827), FADS1 (PIP = 0.632, PP.H4 = 0.942), and ACTR1B (PIP = 0.566, PP.H4 = 0.994) as convergent genes (Figure 5). ABO, LINC00675, and FUT3 reached cTWAS significance but not PP.H4 > 0.80 (Table S3). For ABO, posterior mass concentrated on PP.H3, indicating distinct GWAS and eQTL signals rather than a biological null; this interpretation is compatible with the established ABO blood‐group epidemiology and motivates haplotype‐aware coloc.susie analysis [17]. The LINC00675 and FUT3 discordance may likewise reflect LD or violation of the single‐causal‐variant assumption. Conversely, ABHD12B, ZBTB38, TMBIM1, and CDH1 showed strong colocalization without cTWAS significance and are summarized with expression‐model details in Table S5. MAGMA was an orthogonal, eQTL‐independent cross‐check rather than part of the convergence filter: 45 genes reached Bonferroni significance, but only SMAD9 and FADS1 overlapped the convergent set (Figure 6).

### 3.4. AlphaGenome Reveals Distinct Regulatory Perturbation Signatures for Each CRC Risk Variant

AlphaGenome variant effect prediction for the four lead variants revealed gene‐specific patterns of regulatory perturbation across chromatin accessibility, transcription, and three‐dimensional genome architecture (Figure 7). All four variants produced detectable signals across multiple epigenomic modalities, with RNA‐seq and CAGE exhibiting the most widespread effects.

Figure 7AlphaGenome variant effect prediction for four CRC risk variants. Full‐page panels for (A) ACTR1B (chr2: 97,080,789, A>G), (B) FADS1 (chr11: 61,814,472, T>C), (C) MAP3K2 (chr2:127,304,472, T>C), and (D) SMAD9 (chr13: 36,882,798, C>T). Each panel shows Hi‐C contact maps (reference and delta) predicted across 28 cell types, chromatin interaction arcs (baseline, increased in ALT, decreased in ALT), GENCODE gene track, ENCODE cCRE annotations (promoter, proximal enhancer, distal enhancer, and CTCF/insulator), tissue‐specific H3K27ac ChIP‐seq tracks (sigmoid colon, transverse colon, and HepG2), and AlphaGenome predicted signals with delta tracks (*Δ* = ALT—REF) for DNase‐seq, ATAC‐seq, RNA‐seq, and CAGE. Variant position is indicated by dashed magenta line.

**Figure:**
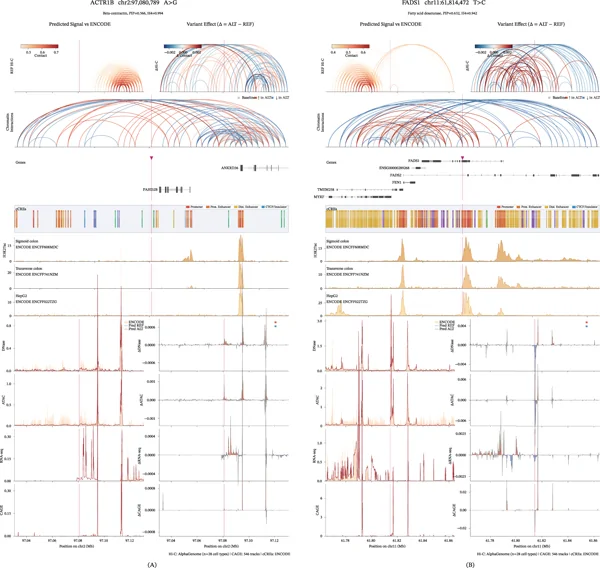
(A)

**Figure:**
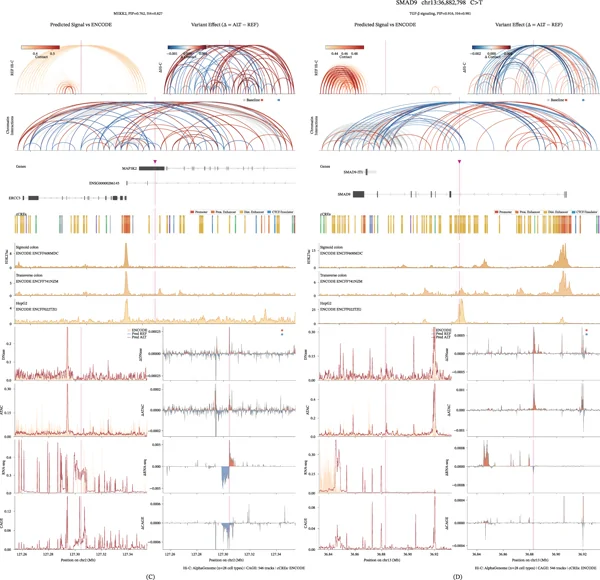
(B)

The *FADS1* variant (chr11: 61814472, T > C) exhibited the strongest overall regulatory impact, with 51.8% of CAGE tracks showing significant perturbation (283 of 546 tracks, |*q* *u* *a* *n* *t* *i* *l* *e* *s* *c* *o* *r* *e*| > 0.90; because track denominators differ markedly across modalities, ranging from 196 ATAC‐seq to 22,148 RNA‐seq tracks, per‐modality percentages are not directly comparable across assays, and the full numerator and denominator counts are provided in Table S1), consistent with the known role of the *FADS* locus as one of the strongest eQTL signals in human tissues [20, 30]. RNA‐seq analysis revealed 2986 significantly affected tracks (13.5% of 22,148 total), indicating broad transcriptional consequences. Hi‐C contact map predictions demonstrated prominent chromatin interaction changes centered on the variant position, with altered looping patterns extending across the *FADS* gene cluster.

The *SMAD9* variant (chr13: 36882798, C > T) primarily affected RNA‐seq signals (424 significant tracks, 4.5%), with relatively modest effects on chromatin accessibility (5.4% of 196 ATAC tracks and 2.3% of 717 DNase tracks) and minimal CAGE perturbation. This pattern suggests that the variant predominantly influences transcriptional elongation or posttranscriptional regulation rather than promoter‐proximal activity, consistent with its intronic location within the *SMAD9* gene body.

*ACTR1B* (chr2: 97080789, A > G) demonstrated the highest colocalization evidence (PP.H4 = 0.994) and exhibited broad but moderate regulatory effects across all four modalities (7.0% for RNA‐seq, 3.6% for ATAC, 3.3% for DNase, and 3.5% for CAGE). Hi‐C contact map analysis revealed a dense network of chromatin interactions at this locus, with 28 cell types showing consistent contact architecture changes. Notably, the variant was positioned near multiple ENCODE candidate *cis*‐regulatory elements (cCREs), including proximal enhancers and CTCF/insulator elements, suggesting a role in modulating chromatin domain boundaries.

*MAP3K2* (chr2:127304472, T>C) exhibited a distinctive pattern characterized by strong RNA‐seq effects (1417 significant tracks, 12.8%) and substantial CAGE perturbation (103 tracks, 18.9%), coupled with the highest DNase‐seq maximum raw score among all four variants (0.123). The delta RNA‐seq track visualization revealed a prominent region of transcriptional downregulation immediately downstream of the variant, overlapping with the *MAP3K2* gene body, suggestive of a *cis*‐regulatory mechanism affecting *MAP3K2* expression (Figure 7).

### 3.5. Western Blot Analysis Shows Reduced Protein Expression in CRC Cells

**Figure 8:**
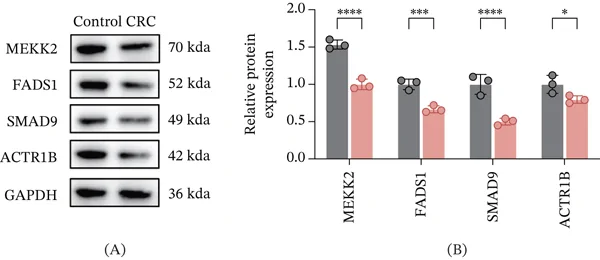
Western blot analysis of prioritized CRC susceptibility gene products. (A) Representative blots for MEKK2 (MAP3K2, approximately 70 kDa), FADS1 (52 kDa), SMAD9 (49 kDa), ACTR1B (42 kDa), and GAPDH (36 kDa). (B) Densitometric analysis normalized to GAPDH. Data are mean plus or minus SD from three independent experiments per cell line.  ^∗^ *p* < 0.05,  ^∗∗∗^ *p* < 0.001,  ^∗∗∗∗^ *p* < 0.0001 versus NCM460; these tumor‐cell measurements are supportive protein‐level evidence rather than a direct test of the germline mechanism.

Western blot analysis compared the four prioritized proteins in CRC cells and NCM460 (Figure 8). All four proteins were lower in the CRC cell lines, which is consistent at the protein level with the negative cTWAS expression direction but does not directly test a germline mechanism. SMAD9 was approximately 45% of the NCM460 level (FDR‐adjusted *p* < 0.0001), MAP3K2 approximately 40% (*p* < 0.0001), FADS1 approximately 65% (*p* < 0.001), and ACTR1B approximately 80% (*p* < 0.05). GAPDH was uniform across samples. Because the measurements derive from established tumor cell lines and a single SV40‐transformed comparison line, they are interpreted as supportive somatic expression evidence.

### 3.6. Cell‐Line Genotypes Are Associated With SMAD9 and FADS1 Protein Abundance

Sanger sequencing showed that HCT116 and SW480 were heterozygous at each assayed variant. RKO was Alt/Alt and NCM460 was Ref/Ref for SMAD9, FADS1, and ACTR1B, whereas the orientation was reversed for MAP3K2 (Table S8). Across the four cell‐line means, alternate‐allele dosage was inversely associated with SMAD9 protein abundance (Pearson r = −0.985, BH‐adjusted *p* = 0.029) and FADS1 abundance (*r* = −0.995, BH‐adjusted *p* = 0.020) (Figure 1A,B). MAP3K2 (*r* = −0.500, BH‐adjusted *p* = 0.667) and ACTR1B (*r* = −0.316, BH‐adjusted *p* = 0.684) showed no significant dosage trend (Figure 1C,D). Because each correlation contains four genetically and biologically distinct cell lines, these analyses are descriptive support for genotype‐expression coupling rather than a population‐level causal estimate.

### 3.7. Reporter and ASE Effects Are Locus Dependent

Reference‐versus‐alternate reporter assays identified the clearest effects at SMAD9 and FADS1 (Figure 2A; Table S9). The SMAD9 T construct reduced normalized luciferase activity by 32% in HCT116 and 28% in SW480 relative to the C construct (both *p* < 0.05), with the same direction in HEK293T. At FADS1, the A construct increased activity by 50% in HCT116 and 42% in SW480 (both *p* < 0.05), again with the same direction in HEK293T. MAP3K2 and ACTR1B reference and alternate constructs did not differ significantly in any tested cell line.

ASE followed the reporter direction within the two responsive loci (Figure 2B). The SMAD9 alternate allele accounted for 0.40–0.44 of corrected allelic mRNA, whereas the FADS1 alternate allele accounted for 0.61–0.66. MAP3K2 and ACTR1B remained near the equal‐expression baseline (alternate proportions 0.49–0.52). Thus, SMAD9 showed concordant genotype‐protein, reporter, and ASE directions. FADS1 showed clear cis‐regulatory activity in reporter and ASE assays, but its reporter/ASE direction differed from the cell‐line protein‐dosage trend, indicating context‐dependent regulation rather than a single linear variant‐to‐protein mechanism. The null MAP3K2 and ACTR1B results define the limits of the tested cell and episomal reporter systems and were retained without selective reporting.

### 3.8. Lower SMAD9 Expression Marks Poorer Outcome in Colorectal Tumors

**Figure 9:**
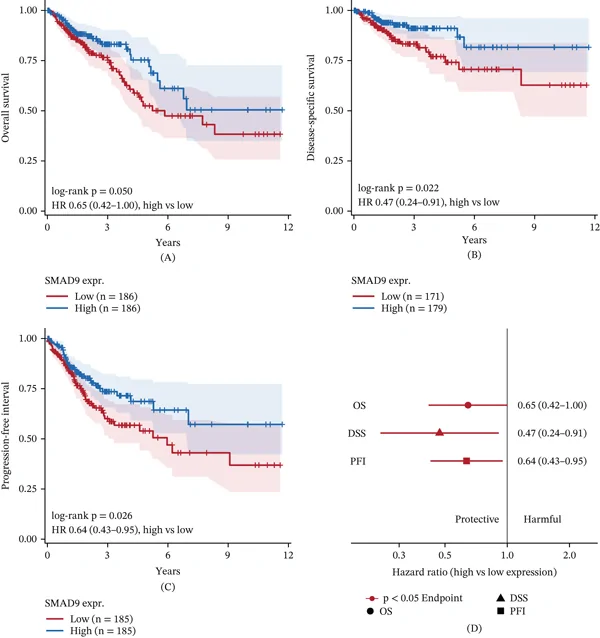
SMAD9 expression and colorectal cancer outcome in TCGA. Kaplan–Meier curves for (A) overall survival, (B) disease‐specific survival, and (C) progression‐free interval in 376 primary tumors split at the median SMAD9 expression, with (D) Cox hazard ratios for high versus low expression and 95% confidence intervals. *p* values are nominal log‐rank tests; none of the SMAD9 endpoints remained significant after Benjamini–Hochberg correction across the 12 gene‐endpoint tests (Table S7).

Expression of the four convergent genes was tested against survival endpoints in the TCGA colorectal cohort, where SMAD9 showed the clearest nominal association (Figure 9). Higher SMAD9 expression was associated with longer overall survival (hazard ratio 0.65, 95% CI 0.42–1.00, log‐rank *p* = 0.050), disease‐specific survival (hazard ratio 0.47, 95% CI 0.24–0.91, *p* = 0.022), and progression‐free interval (hazard ratio 0.64, 95% CI 0.43–0.95, *p* = 0.026). After Benjamini–Hochberg correction across 12 tests, none remained significant (FDR 0.156–0.199; Table S7); the signal is therefore described as suggestive. No other convergent gene showed a consistent prognostic association (Figure S1).

## 4. Discussion

This study integrated causal TWAS, Bayesian colocalization, MAGMA, AlphaGenome prediction, western blot, cell‐line genotyping, genotype‐protein association, ASE, and dual‐luciferase assays to prioritize and functionally interrogate germline CRC susceptibility loci. Four genes met the prespecified convergence criterion: SMAD9, MAP3K2, FADS1, and ACTR1B. The added experiments refined the computational nominations rather than uniformly confirming them: SMAD9 was coherent across all tested layers, FADS1 showed robust but context‐dependent allele‐specific regulation, and MAP3K2 and ACTR1B were null in the tested allelic assays despite their statistical‐genetic support.

SMAD9 (SMAD8) encodes a receptor‐regulated component of BMP/TGF‐beta signaling, a pathway central to intestinal epithelial homeostasis and frequently disrupted in CRC [31, 32]. Its cTWAS and colocalization evidence was reinforced by the most coherent experimental pattern: Increasing rs61947048 T dosage was associated with lower SMAD9 protein, the T reporter construct had lower activity, and T‐containing transcripts were under‐represented in ASE. These results provide direct locus‐level support for a cis‐regulatory mechanism in the tested cell systems. The TCGA association remains nominal after multiplicity correction and should be viewed as hypothesis generating rather than as evidence of a validated prognostic biomarker.

FADS1 encodes a key enzyme in polyunsaturated fatty‐acid metabolism and lies in a strong, pleiotropic cross‐tissue eQTL region [20, 30]. The FADS1 assays detected allele‐specific regulation, but not a simple monotonic chain across modalities: rs1535 A increased reporter activity and allelic mRNA representation, whereas increasing A dosage across the four cell lines was associated with lower protein abundance. This discordance may reflect episomal‐versus‐endogenous context, linked haplotypes within the FADS cluster, or posttranscriptional regulation. It supports regulatory activity at the locus while cautioning against assigning the full cTWAS direction to a single assayed variant.

MAP3K2 (MEKK2) is an upstream regulator of ERK, JNK, and p38 MAPK signaling, a pathway commonly altered in CRC [33]. Its cTWAS PIP and colon‐transverse colocalization support inherited regulation, and AlphaGenome predicted transcriptional and CAGE perturbation at the locus. However, the assayed T>C variant showed neither a genotype‐protein trend nor detectable reporter or ASE imbalance. The result does not negate the locus‐level association; it indicates that the tested variant, fragment, or cellular context did not capture the causal mechanism and prioritizes endogenous perturbation of the credible‐set region for follow‐up.

ACTR1B encodes beta‐centractin, a dynactin component involved in microtubule transport and mitotic‐spindle orientation. Although ACTR1B showed the strongest and most consistent colocalization across tissues, rs7575796 produced no detectable genotype‐protein, reporter, or ASE effect in the tested systems. This null result argues against overinterpreting a single candidate variant and is compatible with a mechanism that depends on chromatin architecture, another variant in the credible set, or an untested cell state.

A strength of the study is that the computational nominations were exposed to several orthogonal experimental tests and that positive, discordant, and null results were retained. The data most strongly support SMAD9 as a cis‐regulated susceptibility locus. FADS1 remains mechanistically plausible but requires haplotype‐aware and endogenous validation, whereas MAP3K2 and ACTR1B remain statistical‐genetic nominations whose tested variants were not functional in these assays. This calibrated interpretation is more informative than treating reduced protein in tumor‐derived cells as direct confirmation of a germline mechanism.

At the computational level, all four convergent genes carried negative cTWAS Z‐scores in every tissue with PIP > 0.10, suggesting that genetically lower expression is associated with higher CRC risk. Their pathways are biologically connected: BMP/TGF‐beta and MAPK signaling interact in colorectal epithelium, FADS1‐derived lipid mediators can modulate proliferative signaling, and ACTR1B‐dependent spindle integrity constrains chromosomal stability. The functional assays nevertheless show that this shared computational direction should not be reduced to an identical single‐variant mechanism at all four loci.

Several limitations warrant consideration. The GWAS analysis was restricted to European ancestry, and the four‐tissue panel did not include other metabolically relevant tissues that may be particularly informative for FADS1. The East Asian lookup provided modest directional support but was not a full multiancestry cTWAS replication (Table S4). NCM460 is SV40 transformed and is not primary normal colonic epithelium. Moreover, genotype‐protein correlations were based on only four cell lines, so genotype is inseparable from cell‐line background and the resulting correlations are descriptive. The ASE and reporter assays used cell models rather than heterozygous patient tissue, and episomal reporters do not reproduce endogenous chromatin. For SMAD9 and FADS1, the experimentally tractable assayed variants also require explicit distinction from the top AlphaGenome‐scored variant at the same locus. AlphaGenome predictions remain computational, cTWAS depends on eQTL prediction models, and coloc.abf assumes one causal variant per locus, an important limitation in the FADS cluster. These points motivate CRISPR‐based endogenous perturbation, primary‐tissue validation, and coloc.susie analysis [17].

Future work should test the complete credible sets with CRISPR interference, activation, or base editing in colonic epithelial models; resolve haplotype effects at FADS1; and repeat genotype‐expression analyses in larger panels and heterozygous primary samples. Single‐cell eQTL and chromatin‐interaction data may identify cell states not represented by the current assays. Multiancestry cTWAS, tissue microarrays, and gene‐perturbation phenotyping will be required before prognostic or therapeutic claims are warranted.

## 5. Conclusions

By integrating cTWAS, colocalization, MAGMA, AlphaGenome, and locus‐directed experiments, we prioritized SMAD9, MAP3K2, FADS1, and ACTR1B as CRC susceptibility genes. The experimental evidence was not uniform: SMAD9 showed concordant genotype‐protein, reporter, and ASE effects; FADS1 showed clear but context‐dependent allele‐specific regulation; and the tested MAP3K2 and ACTR1B variants were null in these systems. These findings refine the inferred mechanisms while preserving the distinction between germline regulation and tumor‐derived expression. The study therefore provides a prioritized, experimentally bounded framework for endogenous and multiancestry validation rather than definitive proof of causality or prognosis.

## Author Contributions

Analytical contributions: C.H., G.Y., and H.X. (co‐first authors) contributed to the conceptualization (analytical design), methodology, software, formal analysis, investigation, data curation, validation, visualization, and writing of the original draft and of the review and editing. Y.X., Z.Y., J.L., P.W., M.L., J.X., Q.L., C.L., W.H., X.P., and L.Z. contributed to the investigation (western‐blot validation), data curation, validation, visualization, and writing of review and editing. Organizational and supervisory contributions: S.C., F.Z., L.D., and S.L. (joint corresponding authors) contributed to the conceptualization (project framing), resources, supervision, project administration, funding acquisition, and writing of review and editing. All authors contributed to the article. C.H., G.Y., and H.X. contributed equally to this work. S.L., L.D., F.Z., and S.C. jointly supervised this work.

## Funding

This study was supported by the Basic and Applied Basic Research Foundation of Guangdong Province (10.13039/501100021171, 2021A1515110519); Guangzhou Basic and Applied Basic Research Foundation (2025A03J4475); Guangzhou Municipal Science and Technology Project (10.13039/501100010256, 2025A04J4692, 2024A03J0485).

## Disclosure

All authors approved the submitted version.

## Conflicts of Interest

The authors declare no conflicts of interest.

## Supporting Information

Additional supporting information can be found online in the Supporting Information section.

## Supporting information


**Supporting Information 1** Figure S1: Prognostic association of all four convergent genes in TCGA colorectal tumors. (A) Cox hazard ratios (high vs. low median‐split expression) for *SMAD9*, *MAP3K2*, *FADS1*, and *ACTR1B* across overall survival, disease‐specific survival, and progression‐free interval. (B–E) Kaplan–Meier overall survival curves for each gene. Among the four genes, only *SMAD9* reached nominal significance, and *MAP3K2*, *FADS1*, and *ACTR1B* showed no consistent prognostic association.

**Supporting Information 2** Figure S2: Functional‐assay design and cross‐assay concordance. (A) Reporter configurations used for enhancer‐like SMAD9/ACTR1B fragments in pGL3‐Promoter and promoter‐like FADS1/MAP3K2 fragments in pGL3‐Basic. (B) Relationship between log2 alternate/reference effects measured by ASE and dual‐luciferase assays. Each point is a summarized locus‐by‐cell comparison included in the concordance analysis.

**Supporting Information 3** Tables S1–S9 (05_Supplementary_Tables_S1‐S9.xlsx): Table S1 reports AlphaGenome perturbed‐track counts across modalities and thresholds; Table S2 reports per‐tissue cTWAS Z‐scores; Table S3 summarizes cTWAS‐significant genes that did not meet the colocalization threshold; Table S4 presents trans‐ancestry association lookups for the four lead variants; Table S5 summarizes colocalization‐only genes; Table S6 reports threshold‐sensitivity analyses; Table S7 reports TCGA‐COADREAD survival analyses with multiplicity correction; Table S8 provides cell‐line genotypes and genotype‐protein associations; and Table S9 provides dual‐luciferase and allele‐specific expression summary values.

## Data Availability

CRC GWAS summary statistics are available from the original study [5]; GTEx v8 eQTL, 1000 Genomes, ENCODE, and AlphaGenome resources are available from their respective portals. Project‐generated summary outputs, analysis code, and the new functional‐assay summary tables must be deposited in a citable archive before submission.
