# Supplementary material for: From Germline Variants to Tumor Outcome: GWAS‐Based Functional Genomics Prioritizes Colorectal Cancer Susceptibility Genes and Links SMAD9 to Prognosis
Source: Hum Mutat. 2026 Aug 20;2026:5617762. doi: 10.1155/humu/5617762 (PMC13492055; doi:10.1155/humu/5617762)
Supplement: Supplementary file 1 [file HUMU-2026-5617762-s001.pdf]

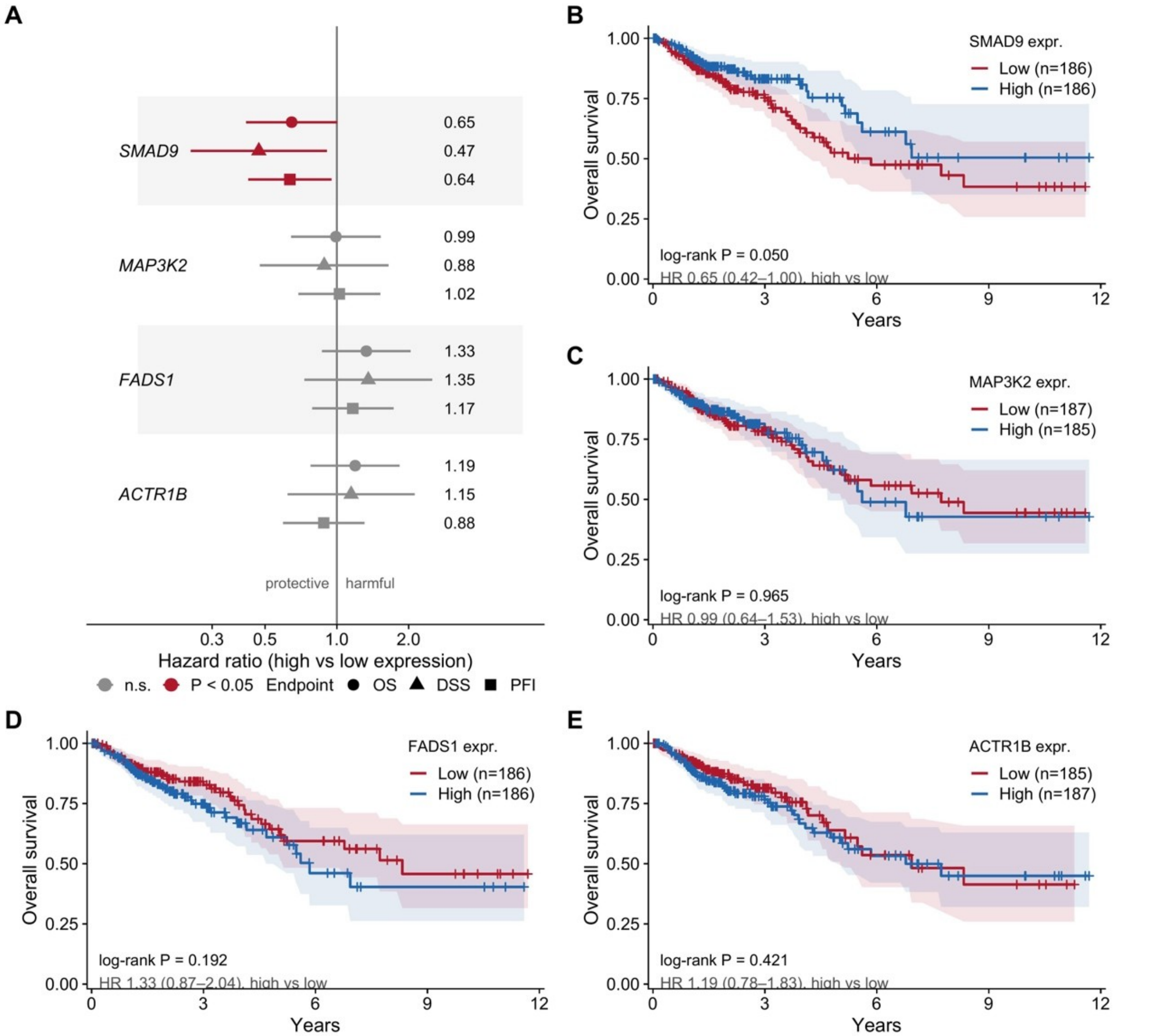
A
SMAD9
0.65
0.47
0.64
MAP3K2
0.99
0.88
1.02
FADS1
1.33
1.35
1.17
ACTR1B
1.19
1.15
0.88
protective
harmful
0.3
0.5
1.0
2.0
Hazard ratio (high vs low expression)
n.s.
P < 0.05
Endpoint
OS
DSS
PFI
B
SMAD9 expr.
Low (n=186)
High (n=186)
Overall survival
log-rank P = 0.050
HR 0.65 (0.42–1.00), high vs low
Years
C
MAP3K2 expr.
Low (n=187)
High (n=185)
Overall survival
log-rank P = 0.965
HR 0.99 (0.64–1.53), high vs low
Years
D
FADS1 expr.
Low (n=186)
High (n=186)
Overall survival
log-rank P = 0.192
HR 1.33 (0.87–2.04), high vs low
Years
E
ACTR1B expr.
Low (n=185)
High (n=187)
Overall survival
log-rank P = 0.421
HR 1.19 (0.78–1.83), high vs low
Years
