# Supplementary material for: From Germline Variants to Tumor Outcome: GWAS‐Based Functional Genomics Prioritizes Colorectal Cancer Susceptibility Genes and Links SMAD9 to Prognosis
Source: Hum Mutat. 2026 Aug 20;2026:5617762. doi: 10.1155/humu/5617762 (PMC13492055; doi:10.1155/humu/5617762)
Supplement: Supplementary file 2 [file HUMU-2026-5617762-s002.pdf]

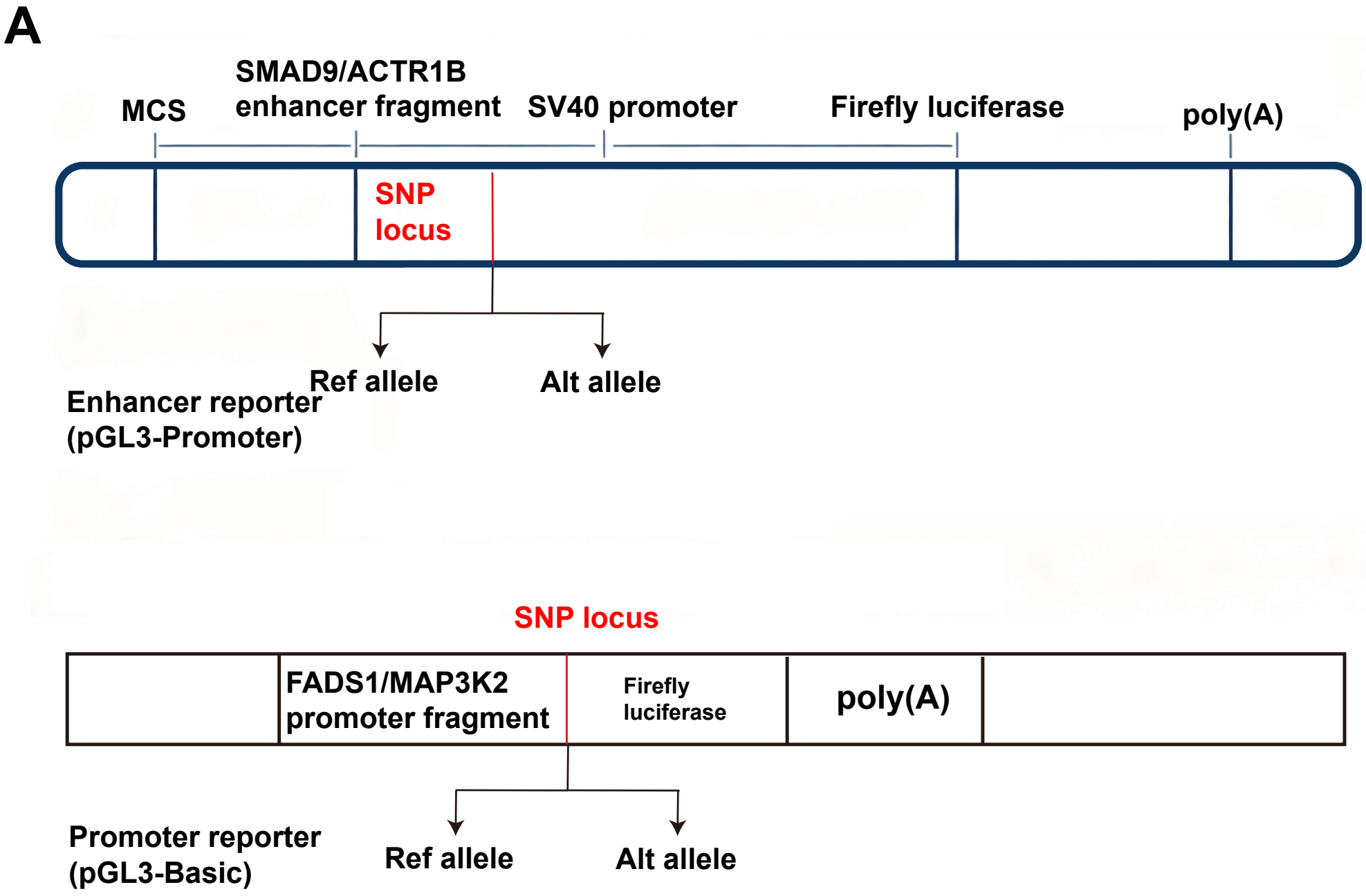

A
MCS
SMAD9/ACTR1B
enhancer fragment
SV40 promoter
Firefly luciferase
poly(A)
SNP
locus
Enhancer reporter
(pGL3-Promoter)
Ref allele
Alt allele
SNP locus
FADS1/MAP3K2
promoter fragment
Firefly
luciferase
poly(A)
Promoter reporter
(pGL3-Basic)
Ref allele
Alt allele

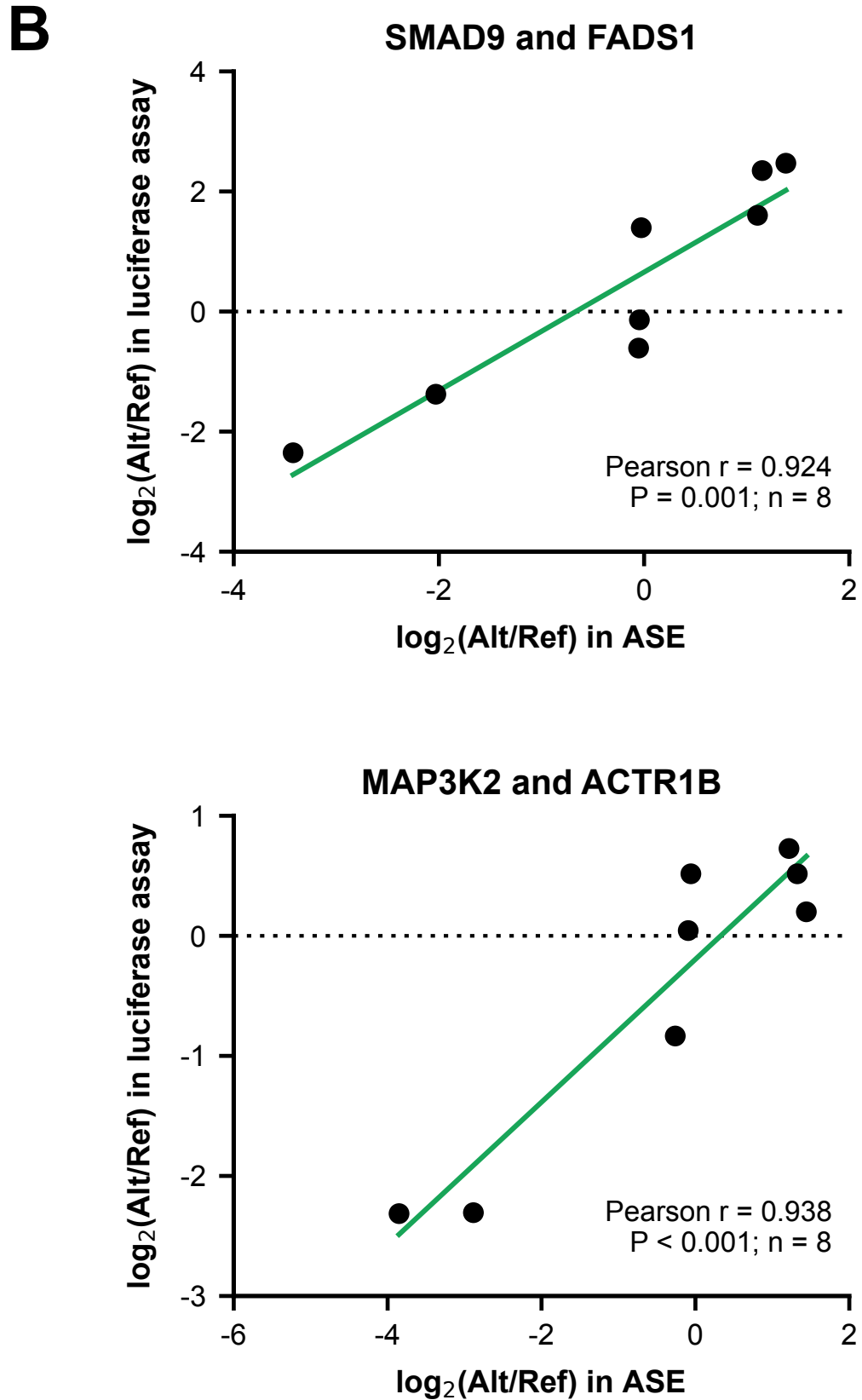

B
SMAD9 and FADS1
log2(Alt/Ref) in luciferase assay
log2(Alt/Ref) in ASE
Pearson r = 0.924
P = 0.001; n = 8
MAP3K2 and ACTR1B
log2(Alt/Ref) in luciferase assay
log2(Alt/Ref) in ASE
Pearson r = 0.938
P < 0.001; n = 8
